# Ultrasound Assessment of Carotid Intima-Media Thickness: Comparison between Diabetes and Nondiabetes Subjects, and Correlation with Serum Vitamin D

**DOI:** 10.1155/2024/7178920

**Published:** 2024-03-15

**Authors:** Sameeah Abdulrahman Rashid

**Affiliations:** Department of Surgery, College of Medicine, Hawler Medical University, Erbil, Iraq

## Abstract

**Methods:**

This multicenter cross-sectional study was performed on two groups of adults (nondiabetes and type 2 diabetes) of various ages, sexes, and body mass index (BMI). CIMT for each side was measured at three segments using high-resolution ultrasound, and the mean of both sides was determined. Comparison was made between each group, and the association of CIMT with each of age, sex, BMI, serum vitamin D status, smoking, and physical activity status was studied. The chi-square test was used to compare categorical data, and binary logistic regression was utilized to ascertain the relationship between CIMT and the study variables.

**Results:**

A significant difference was observed between the CIMT of the diabetes and nondiabetes group, average CIMT was 0.82 ± 0.23 mm vs. 1.12 ± 0.24 mm for the nondiabetes and diabetes group, respectively, with *P* value <0.005. No significant correlation was observed between serum vitamin D level and CIMT neither in the study group as a whole nor in either subgroup; however, a significant association was observed between CIMT with each of age, sex, BMI, smoking, and physical activity status.

**Conclusion:**

Ultrasound is a sensitive tool for CIMT evaluation. Diabetes has a 5.4-fold higher risk of having high CIMT. Serum vitamin D level showed no significant influence on CIMT. Smoking, BMI, and physical activity are among the modifiable risk factors with significant influence on CIMT.

## 1. Introduction

Diabetes is a chronic metabolic disorder with a continuous increase in its prevalence globally [1]. Atherosclerosis is still considered the major cause of death and disability among diabetes, especially in those with type 2 diseases [2], and it is a well-established independent but modifiable risk factor for stroke, both ischemic and hemorrhagic type [3].

Diabetes leads to thickening of the carotid intima-medial layer which is considered an early sign of atherosclerosis hereby causing early structural impairment of the carotid vessels [3], on the other hand, there are reports about the effect of vitamin D deficiency on Carotid Intima-Media Thickness (CIMT), and many studies including randomized clinical trials have studied the effect of vitamin D supplement on CIMT [4–7]; however the results for the correlation between vitamin D and atherosclerosis are still controversial [8].

CIMT measurement is considered an accepted surrogate marker of atherosclerosis, and this measurement can be obtained safely and noninvasively through B-mode ultrasound [9]. An increase in CIMT could also be referred to as an index of atherosclerosis in other vascular beds and can predict future cardiovascular and cerebrovascular events as it is thought to be an adaptive mechanism to cardiovascular stress in situations like diabetes mellitus and hypertension [10].

Studies are continuously carried out to evaluate CIMT in various groups of individuals with variable situations and evaluating its correlation with various factors, however, some results are controversial and with conflicting outcomes. In this study, we aimed to find out the extent of the effect of diabetes on CIMT by comparing it to a nondiabetes control group in a larger study sample and with a different ethnicity than previous works, and we also aimed to explore whether vitamin D status can affect CIMT in either of these groups. These actions could render this study different from previous works and improve the findings of the previously published data.

## 2. Methods

### 2.1. Study Design and Sample

The study is a multicenter cross-sectional, carried out in the Radiology Department of two Teaching Hospitals and Diabetic Center, between February and September 2023. A total of 200 consecutive adult cases were included, including 69 cases with type 2 diabetes mellitus and 131 cases with no diabetes as a control group. Subjects of various ages, sexes, and BMIs were included for each group.

Exclusion criteria include age under 18 years, bodybuilders, type 1 diabetes, pregnant women, and cases with a history of ischemic heart disease, stroke, and chronic renal disease. Subjects with technical difficulties in obtaining proper CIMT were also ruled out from the study.

The sample was divided according to BMI into four subgroups: underweight (<18.5 kg/m^2^), normal (18.5–24.99 kg/m^2^), overweight (25–29.99 kg/m^2^), and obese (≥30 kg/m^2^). The cases were also divided into four age categories (≤44 years old, 45–60 years old, 61–75 years old, ≥76 years old).

BMI was calculated using the standard formula after the weight was measured in kg and the height in meters (m) with the patient standing.

Subjects were considered to have diabetes if they were a known case for the disease and/or were under treatment for at least one year, documented by laboratory results from their medical records (fasting blood sugar level and HbA1c), and validated by endocrinologists.

For serum vitamin D assessment, quantification of 25(OH) D levels (ng/ml) was performed from serum samples using automated immunoassays. Liaison 25(OH) Total Vitamin D Assay DiaSorin Liaison XL (DiaSorin, Italy). The normal range of the test was 19.9–79.3 pg/mL. Cases with serum vitamin D levels below 19.9 pg/mL were considered as low vitamin D.

Information about age, history of cardiovascular disease, stroke, chronic renal disease, smoking, and lifestyle/physical activity was obtained using a standardized questionnaire administered to every participant.

The study was approved by the local ethics committee of our college. The participants were informed about the nature and the aim of the study and informed consent was obtained.

### 2.2. Ultrasound Technique

Each participant underwent an ultrasound evaluation of the carotid artery performed by two expert radiologists with 12- and 15-year experience in the field. Each participant was evaluated once, but measurements of the CIMT for each segment of interest were taken several times and the mean was recorded to reduce the chance of error and minimize intrarater variability of the measurements.

For each side of the neck, measurements were taken from three carotid segments: (a) the common carotid artery (CCA) 1 cm segment proximal to the carotid bulb, (b) the carotid bulb, and (c) 1 cm segment in the internal carotid artery distal to the bulb.

The ultrasound machines used in the study were Philips, HD11 XE Ultrasound System and GE, Voluson S8 equipped with a 7–10-megahertz linear array transducer.

**Figure 1:**
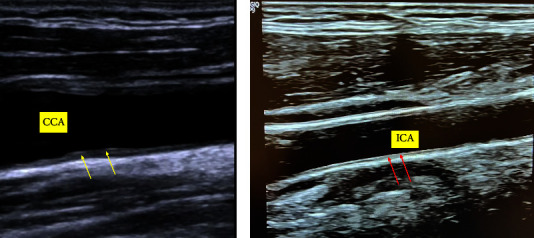
Displaying the CIMT of two different patients. (a) Irregular thickening of the CIMT (yellow arrows) of the far wall of the CCA (common carotid artery) in a 52-year-old diabetic man. (b) Normal smooth CIMT (red arrows) of the far wall of the ICA (internal carotid artery) in a nondiabetic 50-year-old man.

The participants were positioned supine, neck extension was achieved by a pillow under the neck and the head turned to the contralateral side for the side to be assessed. Acoustic gel was applied to reduce the air interface between the transducer and the skin. Scanning and measurement of the carotid vessels were done longitudinally, and once an optimal image was achieved, the image was zoomed and frozen, and the measurements of the carotid IMT were taken, and averaged in millimeters. For each carotid segment, the IMT measurements were taken from the leading edges of the far wall echoes. If a plaque is faced within the segment of interest, it was also included in the measurement. Three measurements of carotid IMT for each side were eventually obtained for each participant, then the mean of these three measurements was assigned as mean right/left CIMT, while the mean of both right and left side was assigned as average CIMT.  Figure 1 displays examples of two cases one with normal and the other with thick CIMT.

### 2.3. Statistical Analysis

Data analysis was carried out using SPSS software version 26.0. Before analysis, the data were checked for normality and outliers. A chi-square test was used to compare categorical data, while an independent *t*-test and one-way ANOVA were used to evaluate quantitative variables. Binary logistic regression was utilized to ascertain the relationship between CIMT with sex, physical activity, DM, BMI, vitamin D state, smoking, and age groups. *P* < 0.05 was used as the statistical significance threshold. To correct biases in the analysis, we employed robust statistical methods, including inverse probability weighting (IPW) and propensity score adjustment. These techniques accounted for potential confounding variables, resulting in a more accurate estimation of the associations under investigation.

## 3. Results

The study sample comprised 200 cases, 69 subjects were diabetes and 131 were nondiabetes (control). Females constituted 47.5% of the whole sample, but they constituted 63.7% in the diabetes group vs. 39% in the control group.

The mean age of the whole study sample was 45.73 ± 13.37, ranging from 24 to 86 years. The mean age of the diabetes group was significantly higher than the control group (58.20 ± 9.09) vs. (42.10 ± 15.40 years) with *P* < 0.001. Most of the subjects, both in the diabetes and control group belonged to the age group ≤60 years (163 cases, 81.5%). The details of the age and other demographic data for each group are shown in Table 1.

The mean BMI of our sample was (23.70 ± 4.63), and the diabetes group displayed significantly lower mean BMI than the control group (*P* < 0.001). The majority of diabetes cases fell in the normal or underweight group for BMI, while the majority of the control group cases fell into the overweight and obese category of BMI, as displayed in Table 1.

Considering vitamin D status, 92 cases (46%) of the sample had normal serum vitamin, among which 38 cases belonged to the diabetes group, with no significant association being observed between vitamin D status and whether being diabetic or not (*P* value =0.062), the detail is shown in Table 1.

The average CIMT of the study sample in general was (0.85 ± 0.29) mm, while the average was (0.82 ± 0.23) mm vs. (1.12 ± 0.24) mm for the nondiabetes and diabetes groups, respectively, with *P* value <0.005 indicating a significant difference in the average CIMT between each group.  Table 2 shows the detail of the mean CIMT for each carotid segment and each side in correlation to age, sex, BMI, and vitamin D status.

It is evident from Table 3 that there is a statistically significant correlation between CIMT and age both in the diabetic and control group, as the average CIMT increased from (0.62 ± 0.12) mm for the group under 45 years to (1.15 ± 0.18) mm for the group over 75 years in the nondiabetic individual vs. (0.81 ± 0.08) to (1.46 ± 0.04) mm in the diabetic group, respectively, while no such association was found between CIMT with each of vitamin D status, BMI, and sex.

Unadjusted odds ratios of (CIMT) were made with diabetes, vitamin D status, sex, activity status, BMI, age, and smoking as shown in Table 4. We observed that the diabetic group was at a 5.40-fold higher risk of having thick CIM than non-DM participants, (*P*=0.001).

Regarding Vitamin D, our observation has shown no statistically significant association between CIMT and vitamin D status (*P* value = 0.102), although individuals with low levels of vitamin D were 1.7 times more likely to have a higher CIMT than those with normal serum vitamin D levels. Men had a 1.56 times higher risk of high CIMT compared to women. The association was statistically significant (*P*=0.001). A similar result was found for activity status (Table 4).

The risk of having thickened carotid intima-media (CIM) was lower in individuals with a lower BMI. Specifically, those with a normal BMI had a 37% lower chance of having thickened CIM than obese participants. In terms of age, the study found that the risk of an increase in CIMT increased with age. Those aged 76 years or older had a 6.57 times higher risk of thickened CIM compared to participants aged 44 years or younger (*P*=0.001). In addition, nonsmokers had a 73% lower chance of having thick CIM compared to smokers, and this association was statistically significant (*P*=0.001). Please refer to Table 4 for more details.

Data from Table 5 reveals unadjusted and adjusted average CIMT according to DM status. In the crude model, participants with DM had a higher average CIMT (1.07 ± 0.25) mm than the non-DM group (*P*=0.001), the result remained significant after adjusting for age and some confounding factors like sex, BMI, physical activity, smoking, and vitamin D status.

## 4. Discussion

There is an alarming increase in the number of people with diabetes mellitus worldwide which may be attributed to genetic susceptibility, aging, and the increased prevalence of obesity [11]. Diabetes is responsible for vascular complications at both macrovascular and microvascular levels and carotid vessel involvement are part of these macrovascular complications [12]. On the other hand, vitamin D deficiency is one of the most common nutritional deficiencies, and there have been observations of the presence of an association between atherosclerosis and this deficiency in many studies [13].

In this study, we aimed to determine the extent of the effect of diabetes on CIMT by comparing with a nondiabetic control group and in a larger study sample than many previous works. The studied group is different from other works in term of ethnicity a factor that could contribute in different results and findings, and we further studied the effect of vitamin D status on CIMT to further enhances new findings in the knowledge with this aspect. These points would make the current study different from others and would likely improve the outcome of the current study.

The average CIMT of the study taking the sample in whole was (0.85 ± 0.29) mm, which is almost similar to the result of Kota et al. [10] which showed a value of (0.840 ± 0.2 mm) in a group of patients' mixture of both diabetic and nondiabetic individuals.

The average CIMT in the subjects was significantly higher in diabetes than in the nondiabetes group, and this relationship remained significant even after adjustment for other variables. The diabetes group was 5.4 times more liable for having high CIMT than non-DM participants, this finding agrees with Kota et al. [10], Bhosale et al. [14], and Bulut et al. [15]. The chronic hyperglycaemia in diabetes induces oxidative stress on the vascular endothelial walls, impairs endothelial function, and allows for monocyte adhesion to the endothelial cells [16], and causes both endothelial and vascular smooth muscle dysfunction [17], these factors explain why diabetes are at higher value of CIMT than nondiabetes.

Considering vitamin D status, there was no significant association between CIMT and having normal or low serum vitamin D level, although cases with low vitamin D were 1.7 folds more likely to have thickened CIM compared with subjects with normal serum vitamin D. This result does agree with Winckler et al. [6] who found no independent association between low serum vitamin D and CIMT in a group of type 2 diabetes subjects. Furthermore, Chen et al., in a subgroup of their systemic review and meta-analysis showed that hypovitaminosis D is linked with a 0.85-fold decrease in the odds of having a higher carotid intima-media thickness [7]. The finding with this concern is not concordance with some cross-sectional studies that showed a positive and independent relationship between vitamin D deficiency and CIMT [18, 19], as serum vitamin D level was found to act as a protective factor against the development of carotid plaque [7].

Zhu et al. attributed the protective effect of vitamin D3 against atherosclerosis to the hypothesis that human cytomegalovirus infection can trigger vascular endothelial cell apoptosis, which is the most important factor for the development and progression of atherosclerosis, and this vitamin causes inhibition of endoplasmic reticulum and mitochondrial apoptosis pathway [20].

The current study observed a significant correlation between CIMT and ages both when taking the study sample in general, or when the subjects were grouped into diabetes and nondiabetes. After adjusting for other variables, age remained a strong predictor for an increase in CIMT; this finding is in concordance with Okafor et al. [16] which showed that age is a strong independent predictor for an increase in CIMT. A systemic review by Van Den et al. [21] concluded the presence of a strong linear relation between age and CIMT in both diabetic and healthy groups, and the relationship was not affected by cardiovascular disease or other risk factors. Similarly, Howard et al. observed the same finding in a large American study of Atherosclerosis Risk in Communities (ARIC) participants [22]. The increase in CIMT with age could be the result of vascular aging which occurs as a consequence of chronic low-grade inflammation, endothelial dysfunction, and increased arterial stiffening due to an increase in matrix metalloproteinase production [23].

The results of the current study indicate a significant difference in CIMT between males and females and various BMIs. This result is consistent with the study conducted by Rashid et al. [9], which demonstrated that an increase in BMI by one unit leads to a 0.009 mm increase in CIMT. Likewise, Edgar et al. [24] attributed the sex correlation with CIMT to the difference in gene expression between men and women, which is responsible for the difference in stroke risk.

Physical activity has also shown a significant association with CIMT, as nonactive subjects had a 1.66-fold higher rate of thickened CIM than active participants. Byrkjeland et al. [25] have demonstrated a beneficial effect of exercise training on the progression of CIMT in patients with type 2 diabetes. Regarding the effect of smoking on CIMT, our results have shown that nonsmokers were at a 73% lower chance of having thick CIM compared to smokers, this finding is in line with the result of Alelyani et al. [26] which stated that smokers exhibit a significant increase in the CIMT. The contents of cigarette smoke and the abundant free radicals in, it both contribute to direct damage to the endothelium, inducing inflammation and leukocyte infiltration, with subsequent development of atheroma and thrombosis [27].

### 4.1. Limitation

The main limitation of our work is the manual measurement of CIMT; semiautomatic or automatic measurement may give more accurate results.

## 5. Conclusion

Diabetes patients are at a 5.40-fold higher risk of having a thick CIM compared with nondiabetes. Cases with low vitamin D are 1.7 times more liable to have thickened CIM than normal although the association is not significant. BMI, smoking, and physical inactivity are among modifiable risk factors with significant influence on CIMT. Despite the promising results, further studies with a greater and more standardized sample size are recommended to confirm these findings. Sonographic assessment of the CIMT is an additional element to suggest the diagnosis of atherosclerosis and possibly help prevent complications related to diabetes.

## Figures and Tables

**Table 1:** Demographics of the study sample in both diabetic and control group.

Variables	Subgroups	Total	Diabetes mellitus		*P* value
			No	Yes	
			Means ± SD/*N* (%)		
Age		45.73 ± 13.37	42.10 ± 15.40	58.20 ± 9.09	<0.001

BMI		23.70 ± 4.63	28.39 ± 6.35	24.17 ± 7.28	<0.001

Gender	Male	105 (52.5)	80 (40)	25 (12.5)	<0.001
	Female	95 (47.5)	51 (25.5)	44 (22)	

Physical activity	In-active	122 (61)	93 (46.5)	29 (14.5)	<0.001
	Active	78 (39)	38 (19)	40 (20)	

Smoker	No	115 (57.5)	99 (49.5)	16 (8)	<0.001
	Yes	85 (42.5)	32 (16)	53 (26.5)	
Vitamin D status	Normal	92 (46.0)	54 (27.0)	38 (19.0)	0.062
	Low	108 (54.0)	77 (38.5)	31 (15.5)	

BMI	Under weight	20 (10)	4 (2)	16 (8)	<0.001
	Normal	65 (32.5)	40 (20)	25 (12.5)	
	Over weight	55 (27.5)	43 (21.5)	12 (6)	
	Obese	60 (30)	44 (22)	16 (8)	

Age groups	≤44	82 (41)	78 (39)	4 (2)	<0.001
	45–60	81 (40.5)	39 (19.5)	42 (21)	
	61–75	30 (15)	9 (4.5)	21 (10.5)	
	≥76	7 (3.5)	5 (2.5)	2 (1)	

*Note*. Data presented as mean ± SD or *N* (%), *P* value by the independent *t*-test and chi-square.

**Table 2:** CIMT of each carotid segment and for each side in correlation to age, BMI, sex, and vitamin D status.

		Nondiabetic						Diabetic					
		Mean ± SD in mm											
		Rt CC	Rt BIF	Rt ICA	Lt CC	Lt BIF	Lt ICA	Rt CC	Rt BIF	Rt ICA	Lt CC	Lt BIF	Lt ICA
Age group	≤44	0.58 ± 0.14	0.64 ± 0.23	0.59 ± 0.12	0.60 ± 0.13	0.69 ± 0.28	0.59 ± 0.12	0.71 ± 0.8	0.82 ± 0.19	0.79 ± 0.13	0.74 ± 0.11	1.12 ± 0.22	0.7 ± 0
	45–60	0.76 ± 0.18	0.98 ± 0.42	0.84 ± 0.28	0.79 ± 0.19	0.87 ± 0.27	0.84 ± 0.37	0.89 ± 0.16	1.38 ± 0.64	0.87 ± 0.22	0.89 ± 0.21	1.26 ± 0.46	0.85 ± 0.16
	61–75	0.97 ± 0.22	1.30 ± 0.61	0.97 ± 0.61	1.0 ± 0.31	1.31 ± 0.47	0.96 ± 0.29	0.91 ± 0.14	1.49 ± 0.61	0.98 ± 0.47	1.0 ± 0.32	1.56 ± 0.61	1.08 ± 0.4
	≥76	0.93 ± 0.19	1.27 ± 0.48	1.17 ± 0.75	0.93 ± 0.15	1.64 ± 0.46	0.95 ± 0.38	1.0 ± 0	2.06 ± 0.42	0.80 ± 0.14	1.2 ± 0.28	2.15 ± 0.35	1.05 ± 0.49
	*P* value	<0.001	<0.001	<0.001	<0.001	<0.001	<0.001	0.082	0.015	0.511	0.072	0.019	0.006

BMI	Under wt	0.85 ± 0.26	1.37 ± 0.9	0.72 ± 0.27	0.83 ± 0.35	0.88 ± 0.56	0.80 ± 0.53	0.86 ± 0.16	1.56 ± 0.91	0.95 ± 0.47	0.93 ± 0.22	1.32 ± 0.37	0.83 ± 0.14
	Normal	0.62 ± 0.19	0.82 ± 0.52	0.70 ± 0.44	0.65 ± 0.17	0.79 ± 0.41	0.68 ± 0.34	0.89 ± 0.11	1.44 ± 0.57	0.92 ± 0.27	0.92 ± 0.29	1.46 ± 0.62	0.93 ± 0.29
	Over wt	0.64 ± 0.19	0.69 ± 0.25	0.68 ± 0.22	0.67 ± 0.17	0.80 ± 0.37	0.67 ± 0.22	0.90 ± 0.15	1.28 ± 0.53	0.83 ± 0.20	0.94 ± 0.21	1.25 ± 0.50	0.93 ± 0.37
	Obese	0.75 ± 0.20	0.88 ± 0.29	0.75 ± 0.25	0.76 ± 0.24	0.86 ± 0.33	0.76 ± 0.24	0.90 ± 0.20	1.34 ± 0.58	0.88 ± 0.27	0.90 ± 0.28	1.36 ± 0.56	0.99 ± 0.30
	*P* value	0.007	0.005	0.821	0.048	0.801	0.380	0.859	0.689	0.778	0.986	0.685	0.460

Sex	Male	0.67 ± 0.21	0.79 ± 0.41	0.70 ± 0.29	0.71 ± 0.22	0.84 ± 0.41	0.73 ± 0.32	0.91 ± 0.15	1.40 ± 0.59	0.91 ± 0.28	0.98 ± 0.32	1.40 ± 0.56	0.93 ± 0.32
	Female	0.68 ± 0.20	0.85 ± 0.39	0.73 ± 0.35	0.68 ± 0.19	0.79 ± 0.31	0.68 ± 0.20	0.87 ± 0.15	1.42 ± 0.69	0.89 ± 0.33	0.89 ± 0.21	1.36 ± 0.52	0.91 ± 0.26
	*P* value	0.958	0.953	0.250	0.701	0.087	0.187	0.586	0.803	0.185	0.429	0.396	0.858

Vitamin D status	Normal	0.68 ± 0.21	0.84 ± 0.39	0.72 ± 0.34	0.72 ± 0.22	0.84 ± 0.36	0.72 ± 0.27	0.90 ± 0.15	1.50 ± 0.73	0.93 ± 0.38	0.93 ± 0.27	1.35 ± 0.52	0.93 ± 0.29
	Low	0.67 ± 0.20	0.79 ± 0.42	0.70 ± 0.29	0.68 ± 0.20	0.81 ± 0.39	0.70 ± 0.29	0.88 ± 0.15	1.32 ± 0.54	0.86 ± 020	0.91 ± 024	1.39 ± 0.56	0.91 ± 0.27
	*P* value	0.444	0.631	0.577	0.092	0.673	0.605	0.782	0.124	0.071	0.467	0.849	0.324

*Note*. Data presented as mean ± SD, test by one-way ANOVA.

**Table 3:** Comparison between mean and average CIMT among both diabetic and nondiabetic groups in correlation to serum vitamin D, age, BMI, and sex.

Variables		Nondiabetic			Diabetic		
		Mean ± SD in mm					
		Mean Rt	Mean Lt	Average	Mean Rt	Mean Lt	Average
Serum vitamin D	Normal	0.75 ± 0.26	0.76 ± 0.24	0.75 ± 0.24	1.11 ± 0.32	1.07 ± 0.31	1.09 ± 0.28
	Low	0.72 ± 0.25	0.73 ± 0.24	0.73 ± 0.23	1.0 ± 0.23	1.07 ± 0.29	1.04 ± 0.22
	*P* value	0.390	0.775	0.429	0.65	0.861	0.116

Age group	≤44	0.61 ± 0.13	0.63 ± 0.13	0.62 ± 0.12	0.77 ± 0.09	0.85 ± 0.08	0.81 ± 0.08
	45–60	0.86 ± 0.24	0.84 ± 0.23	0.85 ± 0.23	1.05 ± 0.25	1.00 ± 0.23	1.02 ± 0.20
	61–75	1.08 ± 0.33	1.09 ± 0.31	1.08 ± 0.27	1.13 ± 0.33	1.21 ± 0.37	1.17 ± 0.31
	≥76	1.13 ± 0.24	1.17 ± 0.26	1.15 ± 0.18	1.46 ± 0.18	1.46 ± 0.04	1.46 ± 0.07
	*P* value	<0.001	<0.001	<0.001	0.026	0.004	0.003

BMI	Under wt	0.98 ± 0.39	0.84 ± 0.47	0.91 ± 0.41	1.12 ± 0.43	1.02 ± 0.19	1.07 ± 0.26
	Normal	0.71 ± 0.31	0.71 ± 0.26	0.71 ± 0.28	1.08 ± 0.26	1.10 ± 0.35	1.09 ± 0.27
	Over wt	0.67 ± 0.19	0.71 ± 0.20	0.69 ± 0.18	1.00 ± 0.22	1.04 ± 0.29	1.02 ± 0.24
	Obese	0.79 ± 0.22	0.79 ± 0.24	0.79 ± 0.21	1.04 ± 0.25	1.08 ± 0.33	1.06 ± 0.25
	*P* value	0.038	0.287	0.100	0.712	0.844	0.876

Sex	Male	0.72 ± 0.25	0.76 ± 0.27	0.74 ± 0.24	1.07 ± 0.29	1.10 ± 0.36	1.09 ± 0.31
	Female	0.75 ± 0.26	0.71 ± 0.20	0.73 ± 0.22	1.06 ± 0.29	1.05 ± 0.26	1.06 ± 0.22
	*P* value	0.605	0.063	0.571	0.928	0.123	0.136

**Table 4:** Unadjusted odds ratios of (CIMT) with diabetes, vitamin D status, sex, activity status, BMI, age, and smoking.

Variables	Subgroups	OR (95% CI)	*P* value
Diabetic	No	1	0.001
	Yes	5.40 (2.73–10.64)	

Vitamin D	Normal	1	0.102
	Low	1.70 (0.90–3.21)	

Sex	Female	1	0.001
	Male	1.567 (0.82–2.95)	

Physical activity	Active	1	0.002
	Nonactive	1.66 (0.87–3.14)	

BMI	Obese	1	0.001
	Over weight	2.08 (0.77–5.63)	
	Normal	0.63 (0.28–1.40)	
	Under weight	0.30 (0.10–0.88)	

Age group	≤44	1	0.001
	45–60	1.44 (0.23–8.75)	
	61–75	5.21 (0.19–27.47)	
	≥76	6.57 (1.58–9.32)	

Smoking	Yes	1	0.001
	No	0.27(0.21–1.12)	

**Table 5:** Unadjusted and adjusted mean of (CIMT) according to diabetes mellitus status.

Model	Diabetes mellitus		*P* value
	No	Yes	
Crude	0.74 ± 0.23	1.07 ± 0.25	0.001
Model I	0.74 ± 0.08	1.07 ± 0.015	0.001
Model II	0.73 ± 0.17	1.05 ± 0.12	0.001

Model I. Adjusted for age. Model II. Further adjusted for sex, BMI, physical activity, smoking, and vitamin D. *P* values were obtained using the independent *t*-test.

## Data Availability

The data that support the findings of this study are available on special request by the journal.
